# Proliferation Rate of Somatic Cells Affects Reprogramming Efficiency*

**DOI:** 10.1074/jbc.M112.403881

**Published:** 2013-02-25

**Authors:** Yongyu Xu, Xiaoyuan Wei, Min Wang, Ru Zhang, Yanbin Fu, Mingzhe Xing, Qiuhong Hua, Xin Xie

**Affiliations:** From the ‡ Laboratory of Receptor-based Bio-medicine, Shanghai Key Laboratory of Signaling and Disease Research, School of Life Sciences and Technology, Tongji University, Shanghai 200092 and; the § National Center for Drug Screening, Stake Key Laboratory of Drug Research, Shanghai Institute of Materia Medica, Chinese Academy of Sciences, Shanghai 201203, China

**Keywords:** Cell Proliferation, Induced Pluripotent Stem (iPS) Cell, Myc, Reprogramming, Small Molecules

## Abstract

The discovery of induced pluripotent stem (iPS) cells provides not only new approaches for cell replacement therapy, but also new ways for drug screening. However, the undefined mechanism and relatively low efficiency of reprogramming have limited the application of iPS cells. In an attempt to further optimize the reprogramming condition, we unexpectedly observed that removing c-Myc from the Oct-4, Sox-2, Klf-4, and c-Myc (OSKM) combination greatly enhanced the generation of iPS cells. The iPS cells generated without c-Myc attained salient pluripotent characteristics and were capable of producing full-term mice through tetraploid complementation. We observed that forced expression of c-Myc induced the expression of many genes involved in cell cycle control and a hyperproliferation state of the mouse embryonic fibroblasts during the early stage of reprogramming. This enhanced proliferation of mouse embryonic fibroblasts correlated negatively to the overall reprogramming efficiency. By applying small molecule inhibitors of cell proliferation at the early stage of reprogramming, we were able to improve the efficiency of iPS cell generation mediated by OSKM. Our data demonstrated that the proliferation rate of the somatic cell plays critical roles in reprogramming. Slowing down the proliferation of the original cells might be beneficial to the induction of iPS cells.

## Teaser

**Background:** The relationship between somatic cell proliferation and reprogramming efficiency remains elusive.

**Results:** Reducing somatic cell proliferation by removing c-Myc or adding anti-proliferative compounds dramatically improves induced pluripotent stem cell generation.

**Conclusion:** Hyperproliferation of somatic cells might be detrimental to reprogramming.

**Significance:** The current study provides new perspectives upon the connection between somatic cell proliferation control and reprogramming.

## Introduction

A breakthrough study from the Yamanaka lab (1) demonstrated that somatic cells such as fibroblasts can be reprogrammed into a state of pluripotency that resembles the embryonic stem cell by viral transduction of 4 embryonic transcriptional factors, including Oct-4, Sox-2, Klf-4, and c-Myc (O, S, K, M). The resulting induced pluripotent stem (iPS)^3^ cells can further differentiate into almost any desired adult cell type, providing a promising new opportunity for patient-specific and disease-specific regenerative medicine (2). However, the expansion of this exciting field has been lagged by ill-defined mechanisms and the extremely low overall efficiency. The use of oncogenes and viral vectors also pose a potential safety threat for the application of iPS technology in therapy. Many efforts have been taken to make iPS cells more amenable for clinical applications by using a reduced number of factors (3, 4), nonintegrating gene delivery approaches (5, 6), cell permeable proteins (7), and small molecule reprogramming boosters (8, 9).

c-*Myc* is an oncogene that has been reported as an important inducer of reprogramming (10). Although its functions are not fully understood, c-Myc is believed to activate pluripotent genes and help to maintain the pluripotent state in ES cells (11). Other functions of c-Myc, such as accelerating the cell cycles, loosing the chromatin structures, and preventing cell senescence (12), have also been proposed to be important for reprogramming. Although c-Myc is not an essential reprogramming factor, its omission has been reported to reduce the frequency of germline transmission in chimeric mice (13).

In an attempt to further optimize the reprogramming condition, we observed that removing c-Myc from the OSKM combination reduced the proliferation rate of transduced MEFs, but greatly enhanced the generation of iPS cells. This surprising finding suggested an inverse correlation between the proliferation rate of somatic cells and the overall reprogramming efficiency. Despite rapid progress in the field of reprogramming research, the role of cell cycle control and proliferation of the originating cells are rarely addressed and characterized. Previous studies indicated that somatic cells in a proliferative state responded better to reprogramming factors, and c-Myc played a central role in maintaining such a state (14). However, it has been noticed that under certain defined circumstances, omitting the c-Myc from the reprogramming mixture resulted in higher efficiency (15). A recent study also demonstrated that serum starvation-induced cell cycle synchronization facilitates human somatic cells reprogramming (16). Although the study did not focus on the proliferation of the somatic cells, it is well known that serum starvation will lead to reduced growth in many types of cells.

In this report, we found c-Myc-induced hyperproliferation of MEFs was detrimental to the overall efficiency of reprogramming. Removing c-Myc from the mixture or adding cell cycle inhibitors at the early stage of the reprogramming increased the induction efficiency of iPS cells. The iPS cells obtained without c-Myc were of high quality and capable of producing full-term mice through tetraploid complementation.

## MATERIALS AND METHODS

### 

#### 

##### Chemicals

All chemicals were purchased from Sigma and applied at the indicated concentrations: Nutlin-3 (10 μm), Caylin-1 (10 μm), Aphidicolin (600 nm), Cisplatin (300 nm), Alosine A (100 nm), Compound 52 (100 nm), and Cdk 9 Inhibitor II (100 nm).

##### Retroviral-mediated iPS Cell Generation

Generation of mouse iPS cells using pMXs retroviral vectors containing cDNAs of mouse *Oct-4*, *Sox-2*, *Klf-4*, and c-*Myc* were as described (17). Briefly, MEFs carrying an Oct4-GFP reporter were isolated from OG2 mice and cells from passage 1 to 7 (mostly passage 1 unless otherwise stated) were used for reprogramming (17). Two days (day 2) after viral infection (day 0), MEFs were reseeded at a density of 5000 cells/well onto 96-well plates pre-seeded with irradiated MEF feeders, supplemented with mES medium (DMEM supplemented with 15% FBS, 2 mm l-glutamax, 0.1 mm nonessential amino acids, 0.1 mm β-mercaptoethanol, 1000 units/ml of LIF, 100 units/ml of penicillin, and 100 μg/ml of streptomycin). At day 6, culture medium was replaced with knock-out serum replacement medium (knock-out DMEM supplemented with 15% knock-out serum replacement, 2 mm l-glutamax, 0.1 mm nonessential amino acids, 0.1 mm β-mercaptoethanol, 1000 units/ml of LIF, 100 units/ml of penicillin, and 100 μg/ml of streptomycin).

For serial dilution studies, virus encoding each one of the four Yamanaka factors (O, S, K, and M) was subjected to 5-fold serial dilutions (including zero concentration). For chemical treatment, cells were exposed to various small molecules for 5 days starting from day 3 post-infection. GFP^+^ colonies were photographed and counted using an Olympus IX71 fluorescent microscope equipped with Image Pro Plus software. GFP^+^ colonies were also trypsinized and the GFP^+^ cell number was analyzed using a Guava EasyCyte 8HT flow cytometer.

##### Alkaline Phosphatase Staining and Immunostaining

Alkaline phosphatase staining was performed using a leukocyte AP kit (Sigma, catalog number 85L3R) according to the manufacturer's protocol. For immunofluorescent staining, cells were fixed with 4% paraformaldehyde and incubated with primary antibodies against mSSEA-1 (Santa Cruz, sc-21702) or mNanog (Millipore, AB5731), followed by the appropriate secondary antibodies conjugated to Alexa Fluor 555 (Invitrogen). Nuclei were counterstained with Hoechst 33342 (Sigma). Images were taken with an Olympus IX71 inverted fluorescent microscope or an Olympus FV10i confocal microscope. As to the surface staining for SSEA-1, cells were collected by trypsinization and washed twice on ice with PBS containing 1% FBS. About 5 × 10^5^ cells were then incubated with primary antibody against SSEA-1 (Santa Cruz, sc-21702, 1:50) on ice for 30 min. After two washes with PBS containing 1% FBS, cells were resuspended in allophycocyanin-conjugated anti-mouse secondary antibody (Invitrogen, M31505, 1:10) for 1 h at 4 °C in the dark. Finally cells were washed once in PBS and applied for flow cytometry analysis. The Oct4-GFP-positive cells were gated to further analyze their surface expression of SSEA-1.

##### Chimera Production, Germline Transmission, and Tetraploid Complementation

For production of chimeric mice, zygotes were isolated from superovulated female ICR mice and iPS cells were injected into the resulting blastocysts. Chimeras were produced by implantation of the injected blastocysts into pseudopregnant ICR mice. The chimeras were crossed with ICR mice to confirm the germline transmission. The generation of mice by tetraploid embryo complementation was carried out as previously described (18). In brief, two-cell embryos were collected from oviducts of ICR females, electrofused to produce one-cell tetraploid embryos that were then cultured in KSOM media (Millipore). Ten to fifteen iPS cells were injected into each tetraploid blastocyst and transferred to ICR pseudopregnant recipient females.

##### Real-time PCR

Total RNA was isolated using TRIzol reagent (Invitrogen). About 1.5 μg of RNA was used to synthesize cDNA using Random Hexamer Primer and Moloney murine leukemia virus reverse transcriptase (Promega) according to the manufacturer's protocol. Real-time PCR was performed on a Stratagene Mx 3000P thermal cycler using JumpStart^TM^ TaqReady Mix^TM^ (Sigma, D7440) supplemented with Eva Green (Biotium). For semi-quantitative PCR analysis, the cDNA solution was amplified for 45 cycles at an optimal annealing temperature. Primer sequences are listed in Table 1.

**TABLE 1:** **Primers used for quantitative RT-PCR**

Genes	Sequences
β-*Actin*	GGCTGTATTCCCCTCCATCG
	CCAGTTGGTAACAATGCCATGTT
*Cdk1*	GCTCGTTACTCCACTCCGGT
	TCCACTTGGGAAAGGTGTTCTT
*Cdk2*	TCTCACGGGCATTCCTCTTC
	CCAAAGGCTCATGCTAGTCCA
*Cdk4*	CAATGTTGTACGGCTGATGGAT
	CCGCTTAGAAACTGACGCATT
*Cdk6*	CCACCCCTGTGGACCTCTG
	ATGGGTTGAGCAGATTTGGAAT
*CycA2*	ACCCTGCATTTGGCTGTGAA
	CTGCTTCTTGGAATAGGTATCGTC
*CycB1*	GTGACGTAGACGCAGATGATGG
	ATTTCATCTGAACCTGTATTAGCCA
*CycB2*	GCTGCCTCTTGCCTGTCTCA
	GATGAACTTGGTACGGTTGTCATT
*CycD1*	TGAGGAGCAGAAGTGCGAAGA
	GCCGGATAGAGTTGTCAGTGTAGA
*CycD2*	TGTGCATTTACACCGACAACTCT
	GCACAGAGCGATGAAGGTCTG
*CycE1*	ACCTTTCAGTCCGCTCCAGTA
	TCCAGGGCTGACTGCTATCC
*CycE2*	CTGCTGCCGCCTTATGTCA
	CTTCATTCAGCAAAGCCCAATA
*Plk-1*	TCCCTATTACCTGCCTCACCA
	ACTCAATGGCCTCATTTGTCTC
*Cdc25B*	TCCTGGATAGTGACCACCGT
	GCCCGCCTTCATACTCATAG

##### Cell Proliferation, Cell Death, and CFSE Assay

The cell proliferation assay was performed by counting cell numbers at the indicated time points during the cell culture using flow cytometry. Only Oct4-GFP positive cells were counted when the proliferation rate of iPS cells was measured. To estimate the percentage of cell death, transduced MEFs were harvested by trypsinization and stained with propidium iodide at the concentration of 2.5 μg/ml for 15 min and positive cells were measured by FACS. For the carboxyfluorescein diacetate succinimidyl ester (CFSE) assay, MEFs were seeded at a density of 5 × 10^5^ cells per well in a 6-well plate 1 day prior to staining. Cells were washed twice with PBS and labeled with 1 μm CFSE in PBS for 15 min at room temperature, and then washed three times with cell culture medium to terminate the reaction. Cells were then returned to the CO_2_ incubator for further culture. Cells were harvested at the indicated time points and analyzed by flow cytometry.

##### Statistical Analysis

All data were expressed as mean ± S.E. and analyzed using two-tailed Student's *t* test. *p* < 0.05 was considered statistically significant.

## RESULTS

### 

#### 

##### Removal of c-Myc, but Not Oct-4, Sox-2, or Klf-4, Dramatically Improves the Reprogramming Efficiency in MEFs

**FIGURE 1.**
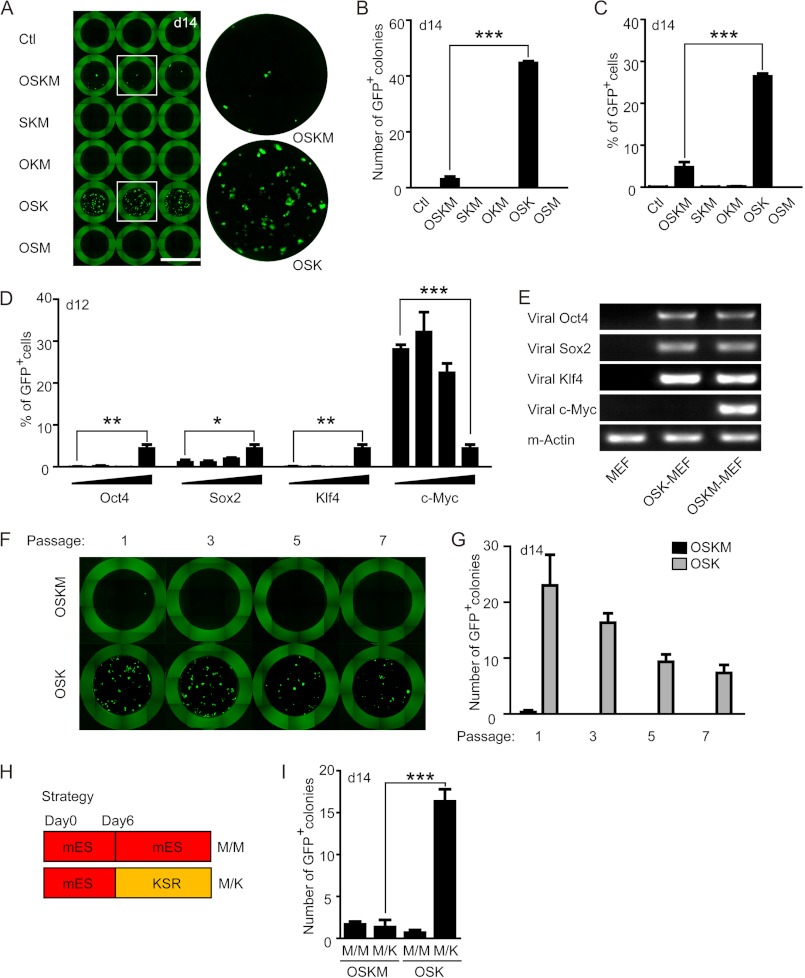
**c-Myc omission from the mixture of Yamanaka boosts iPS cell generation.** *A*, representative image of iPS (GFP^+^) colonies generated from 5000 MEFs infected with various combinations of Yamanaka factors. Uninfected MEFs served as negative control. The *boxed areas in the left column* are presented enlarged at the *right. Scale bars*, 7 mm. *B* and *C,* statistical analysis of the number of GFP^+^ colonies (*B*) and the percentage of GFP^+^ cells (*C*) per well shown in *A. D,* MEFs were infected with 4 factors with one of the factors in serial dilutions, and the percentage of GFP^+^ cells were analyzed by FACS. *E*, PCR analysis to confirm the absence of c-*Myc* in OSK-transduced MEFs. Uninfected MEFs served as a negative control and β-*Actin* was used as a loading control. *, *p* < 0.05; **, *p* < 0.01; ***, *p* < 0.001. *F* and *G,* representative image (*F*) or statistical analysis (*G*) of iPS (GFP^+^) colonies generated from MEFs with different passages infected with 3 or 4 factors. *H,* strategies employed in iPS cell generation. *I,* the number of GFP^+^ colonies generated with OSKM- or OSK-transduced MEFs in M/M or M/K conditions. ***, *p* < 0.001.

In an attempt to optimize the current 4-factor based protocol for iPS cell generation, random combinations of three Yamanaka factors (O, S, K, M) were tested in the reprogramming of OG2 MEFs, which harbor an Oct4-driven GFP reporter (19). In 4-factor-transduced MEFs, 3–5 GFP^+^ colonies were observed at day 14 in each well of a 96-well plate. Unexpectedly, MEFs transduced with OSK showed a robust increase in the number of GFP^+^ colonies, which reached ∼45 in each well, indicating a more than 10-fold increase of the reprogramming efficiency compared with the 4-factor-transduced MEFs (Fig. 1, *A* and *B*). Other combinations of 3 factors resulted in no GFP^+^ colony formation, indicating that removal of O, S, or K is detrimental to reprogramming (Fig. 1, *A* and *B*). FACS analysis also revealed that removing c-Myc from the 4 factors greatly increased the percentage of GFP^+^ cells (Fig. 1*C*). To confirm this observation, 5-fold serial dilutions of individual viral factors were used in iPS cell generation. As shown in Fig. 1*D*, only dilution of the c-*Myc* virus markedly augmented the reprogramming efficiency, whereas the reduction of other factors diminished the reprogramming efficiency. RT-PCR analysis also confirmed the absence of viral *c-Myc* expression in MEFs transduced with OSK (Fig. 1*E*). Because the initial experiments were carried out with first passage MEFs that may contain some stem cells from the embryo, we tested the reprogramming efficiency using MEFs with serial passages. Although the overall reprogramming efficiency decreased when using late-passage MEFs, which was consistent with the notion that cellular senescence is a roadblock of reprogramming (20), omission of c-Myc continued to enhance the generation of iPS colonies (Fig. 1, *F* and *G*).

Because many previous studies reported that 4 factors lead to higher reprogramming efficiency than 3 factors (4, 21), we carefully compared our culture condition with these studies. Many of these earlier studies utilized serum-based medium (mES medium) during the whole reprogramming process (M/M protocol, Fig. 1*H*), whereas our protocol was based on an optimized approach in which the knock-out serum replacement-based medium were applied at day 6 (M/K protocol, Fig. 1*H*). In agreement with previous studies, reprogramming of both OSK and OSKM-transduced MEFs were highly inefficient in the M/M protocol (15, 22), and c-Myc omission led to even lower numbers of GFP^+^ colonies (Fig. 1*I*). However, in the M/K protocol, OSK induced robust generation of GFP^+^ colonies (Fig. 1*I*). So the medium content, especially serum, seems to affect the choice of reprogramming factors.

##### iPS Cells Generated without c-Myc Are Highly Pluripotent

**FIGURE 2.**
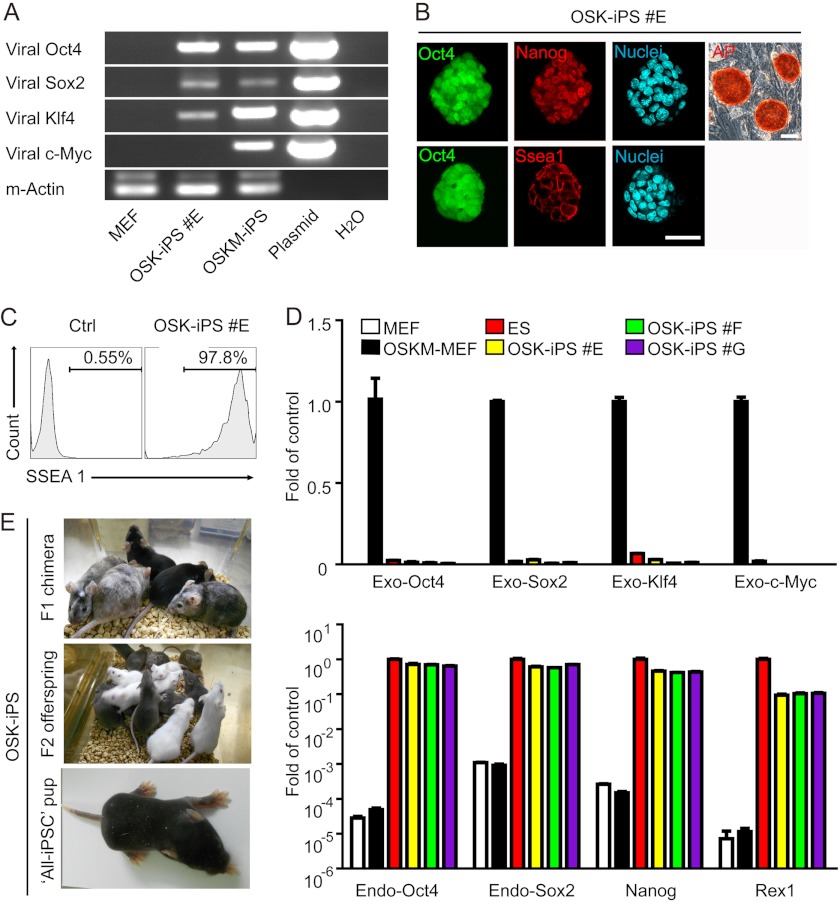
**Pluripotency of iPS cells generated without c-Myc.** *A*, genomic PCR analysis to confirm the absence of c-*Myc* integration in OSK-iPS colonies. Corresponding plasmids were used as positive control, whereas uninfected MEFs were used as negative control. *B*, alkaline phosphatase staining, GFP expression, and immunofluorescent staining of pluripotency markers SSEA-1 and Nanog in OSK-iPS clones. *Scale bars*, 50 μm. *C,* representative histogram from FACS analysis of surface SSEA-1 on three OSK-iPS clones. Cells stained with secondary antibody only served as control. *D*, quantitative PCR analysis of pluripotency genes and exogenous transgenes in OSK-iPS clones. mES cell line E14, MEF, and MEF infected with 4F for 4 days were used as controls. *E*, representative photos of chimeric mice generated from OSK-iPS cells and the agouti coat colored offspring of one male chimera.

A recent study suggested that c-Myc transduction is required for high-quality iPS cell induction (23). Therefore, we thoroughly evaluated the pluripotency of OSK-iPS cells. Two weeks after OSK transduction, GFP^+^ colonies that were morphologically similar to ES cells were picked and expanded. Genomic PCR confirmed the absence of viral c-*Myc* insertion in OSK-iPS clones (Fig. 2*A*). These iPS cells maintain GFP^+^ and ES-like morphology, and express typical pluripotency markers, including Nanog, SSEA1, and alkaline phosphatase (Fig. 2*B*). FACS analysis revealed that more than 97% of the GFP^+^ cells were also SSEA-1 positive, indicating the homogeneity and high quality of the selected clones (Fig. 2*C*). Quantitative RT-PCR analysis confirmed the reactivation of endogenous *Oct4*, *Sox2*, *Nanog*, and *Rex1* and silencing of viral genes in these OSK-iPS clones (Fig. 2*D*). Injection of the OSK-iPS cells into the blastocysts from ICR mice gave rise to high-grade chimeras with a great capacity for germline transmission (Fig. 2*E* and Table 2). One OSK-iPS clone (#F) even passed the most stringent tetraploid complementation assay and supported the generation of an “all-iPS” mouse, demonstrating that the OSK-iPS cells are genuinely pluripotent.

**TABLE 2:** **Characterization of OSK-iPS in chimera formation and germline transmission** All three OSK-iPS cell lines exhibit high capacity in chimera contribution and germline transmission.

OSK-iPS cell line	Blastocytes transplanted	Offspring	Chimera	Total F2 number (with dark coat)	Germline transmission
			%		%
#E	32	9	8 (88.9%)	28 (28);	79.8
				14 (14);	
				30 (30);	
				27 (7)	
#F	20	5	5 (100%)	20 (12);	73.12
				19 (2);	
				26 (26);	
				28 (28)	
#G	50	8	6 (75%)	20 (20);	100
				17 (17);	
				12 (12)	

##### MEFs without c-Myc Transduction Display a Slower Proliferation Rate at the Initial Stage of Reprogramming

**FIGURE 3.**
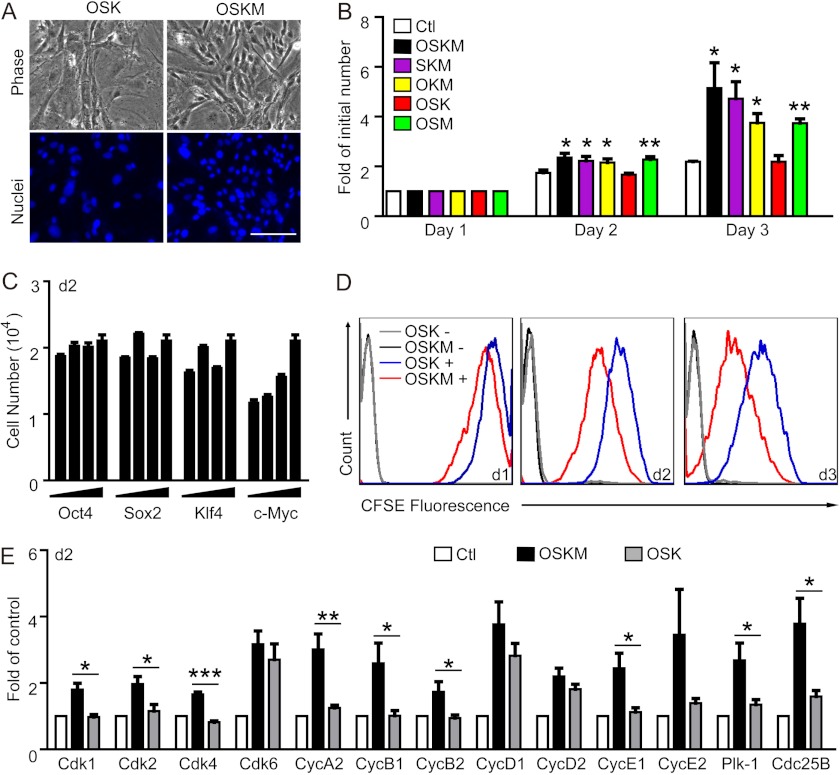
**MEFs without c-Myc overexpression display a slower proliferation rate at the initial stage of reprogramming.** *A,* representative images of MEFs 2 days after the viral infection of 3 or 4 factors. Nuclei were visualized with DAPI staining (*blue*). *Scale bars*, 50 μm. *B,* proliferation rates of MEFs transduced with various combinations of factors at the initial stage of reprogramming (day 1–3) (*, *p* < 0.05; **, *p* < 0.01). *C,* the effect of each factor on MEF proliferation. MEFs were infected with 4 factors with one of the factors in serial dilutions, and the number of MEFs was analyzed at day 2. *D,* proliferation of MEFs infected with 3 or 4 factors monitored using CFSE labeling. CFSE histograms from one representative experiment is shown (*n* = 3). *E,* quantitative PCR analysis of the expression levels of the core components of the cell cycle machinery in MEFs 2 days after the infection of 3 or 4 factors (*, *p* < 0.05; **, *p* < 0.01; ***, *p* < 0.001). The expression level of each gene was normalized to that of β-*Actin* in the same sample and then normalized to MEFs without viral infection.

The role of c-Myc in driving cell proliferation has been well recognized (24, 25). We also observed an enhanced proliferation of MEFs transduced with OSKM compared with MEFs transduced with OSK at the initial stage of reprogramming. Two days after viral infection, density of the cells was clearly higher in MEFs with c-Myc overexpression (Fig. 3*A*). Analysis of viable cell counts also revealed that only the MEFs infected with OSK displayed a similar growth rate as the MEFs without infection, whereas MEFs infected with other combinations of factors containing c-Myc displayed significantly faster growth rates than the control even at 1 day after infection (Fig. 3*B*). In a 5-fold serial dilution assay, only c-Myc stimulated MEF proliferation in a dose-dependent manner (Fig. 3*C*). CFSE analysis also demonstrated that c-Myc overexpression led to an enhanced proliferation of MEFs and hence the left shift of the fluorescent peak (Fig. 3*D*). Consistent with the function of c-Myc as a master transcription regulator for proliferation control, OSKM-transduced MEFs displayed significantly elevated expression of genes encoding many essential members of the cell cycle machinery including cyclin-dependent kinases (Cdk), Cyclins (Cyc), Polo-like kinase 1 (Plk-1), and Cdc25B, whereas OSK-transduced MEFs shared a similar expression profile with uninfected MEFs with the exception of *Cdk6* and *cyclin D1*/*2* (Fig. 3*E*). Collectively, these data indicate that the different proliferation rate of MEFs at the initial stage of reprogramming is one of the most obvious differences regulated by the transduction of various factors, and such a difference might play an important role in the successful reprogramming of somatic cells.

##### Higher Proliferation Rate of MEFs at the Initial Stage Correlates with Lower Reprogramming Efficiency

**FIGURE 4.**
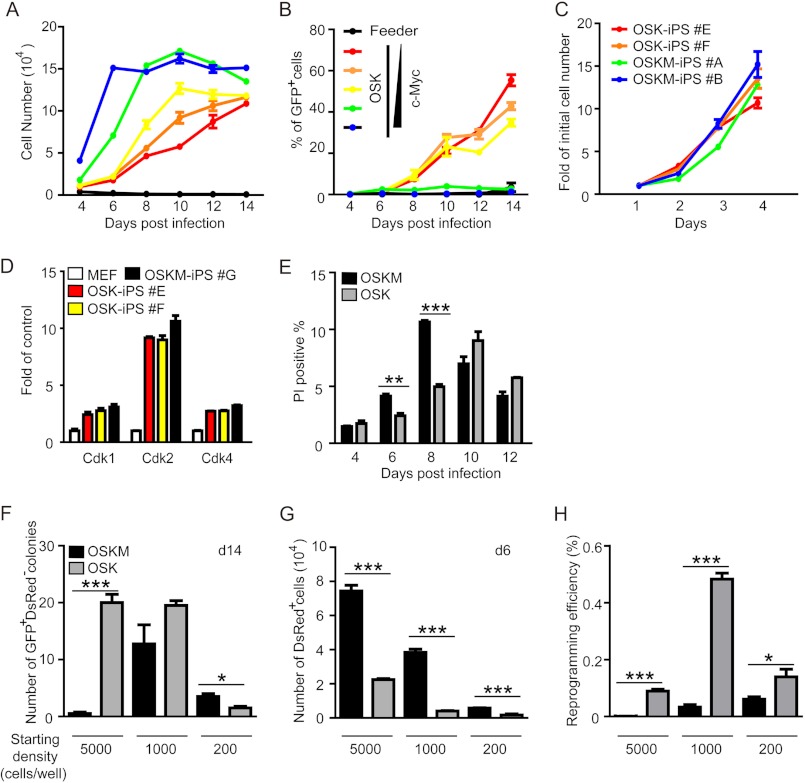
**Higher proliferation rate of MEFs correlates with lower reprogramming efficiency.** *A* and *B,* time course study of the proliferation rate of MEFs and the reprogramming efficiency with OSK and serially diluted c-*Myc* viruses. MEFs were transduced with OSK plus c-Myc in 5-fold serial dilutions (including zero concentration). The number of total cells (*A*) and the percentage of GFP^+^ cells (*B*) were monitored every other day during the reprogramming process. *C,* proliferation rates of two OSK-iPS and two OSKM-iPS clones. Cell numbers were normalized to the value obtained on day 1. *D,* quantitative PCR analysis of the expression levels of *Cdk 1*/*2*/*4* in OSK or OSKM iPS clones. The expression level of each gene was normalized to that of β*-actin* in the same sample and then normalized to MEFs. *E,* characterization of cell death in OSK- or OSKM-mediated reprogramming using propidium iodide staining and FACS analysis. *F* and *G*, MEFs were infected with OSKM or OSK in combination with a Moloney murine leukemia virus 5′ LTR promoter-driven dsRed virus and seeded at different densities in a 96-well plate, the number of GFP^+^ dsRed^−^ colonies were counted at day 14 (*G*), the number of dsRed^+^ cells were recorded at day 6, and the reprogramming efficiency (*H*) was calculated (number of GFP^+^ dsRed^−^ colonies/number of dsRed^+^ cells). *, *p* < 0.05; **, *p* < 0.01; ***, *p* < 0.001.

The correlation between cell-cycle progression and differentiation has been well studied (26, 27). It is generally accepted that cell-cycle arrest might be necessary for differentiation, although not sufficient. Compared with differentiation, reprogramming is also a type of alteration of the cell fate. So we speculated that cell-cycle and growth arrest might also be necessary for somatic cell reprogramming and c-Myc-mediated hyperproliferation might be detrimental to iPS cell generation. Serial dilutions of the *c-Myc* virus were used as tools to control the proliferation rates of MEFs. By counting the total cell number and the percentage of GFP^+^ cells every other day during the reprogramming, we were able to monitor the growth of MEFs and reprogramming efficiency simultaneously. As shown in Fig. 4*A*, the increment in c-*Myc* viral concentrations correlated well with an increase of the proliferation rate of MEFs during the early stage of reprogramming (before day 8). Interestingly, corresponding analysis of GFP^+^ cells revealed an inverse correlation between the initial proliferation rate of MEFs and their reprogramming efficiency, with MEFs transduced with OSK generating the highest percentage of GFP^+^ cells (Fig. 4*B*). Although MEFs transduced with OSK grew much slower than MEFs transduced with OSKM, iPS cells generated with OSK expanded at a similar rate as iPS cells were generated with OSKM, indicating silencing of the exogenous c-*Myc* gene and a reset of cell-cycle control after reprogramming (Fig. 4*C*). Real-time PCR analysis also showed that the expression levels of *Cdks* were similar in OSK- and OSKM-iPS cells, but much higher than in MFEs (Fig. 4*D*). These results indicate that growth arrest might be necessary for reprogramming and hyperproliferation of somatic cells might be detrimental to iPS cell generation.

Because MEFs transduced with OSKM reached confluence at day 6, we wondered whether massive cell death occurred and thus prevented the reprogramming. We counted dead cells by propidium iodide staining and FACS analysis. As shown in Fig. 4*E*, at days 6 and 8, 4F-MEFs did experience more cell death than the 3F-MEFs, but the overall percentage was not more than 10%. So there was no massive cell death in 4F-MEFs. After day 8, the death rate was actually reduced with possible establishment of a new balance. So cell death is not likely the main contributor to the overall low efficiency of 4F-mediated reprogramming.

We then reduced the starting cell density from 5000 to 1000 or 200 cells/well. We also introduced a Moloney murine leukemia virus 5′ LTR-driven dsRed plasmid into the mixture to monitor the proliferation of viral transduced MEFs. Because true iPS cells silence the viral promotor, only the GFP^+^ dsRed^−^ colonies were considered as iPS colonies. When GFP^+^ dsRed^−^ colonies were counted at day 14, we observed that with high starting cell density (5000 cells/well), more iPS colonies were induced by OSK; with medium starting cell density (1000 cells/well), similar numbers of iPS colonies were induced by OSK or OSKM; but with low starting cell density (200 cells/well), more iPS colonies were induced by OSKM (Fig. 4*F*). But because GFP^+^ cells only started to appear at around day 6, the expansion of the transduced MEFs (dsRed^+^ cells) before that must be taken into account. As shown in Fig. 4*G*, MEFs infected with OSKM expanded much faster than MEFs infected with OSK at any seeding density, providing more starting cells for the 4F-mediated reprogramming. So if the actual number of starting cells were normalized (Fig. 4*H*, number of GFP^+^ dsRed^−^ colonies/number of dsRed^−^ cells), OSK-transduced MEFs had better reprogramming efficiency regardless of the seeding cell density.

##### Small Molecule Inhibitors of Cell Proliferation Improve OSKM-mediated Reprogramming

**FIGURE 5.**
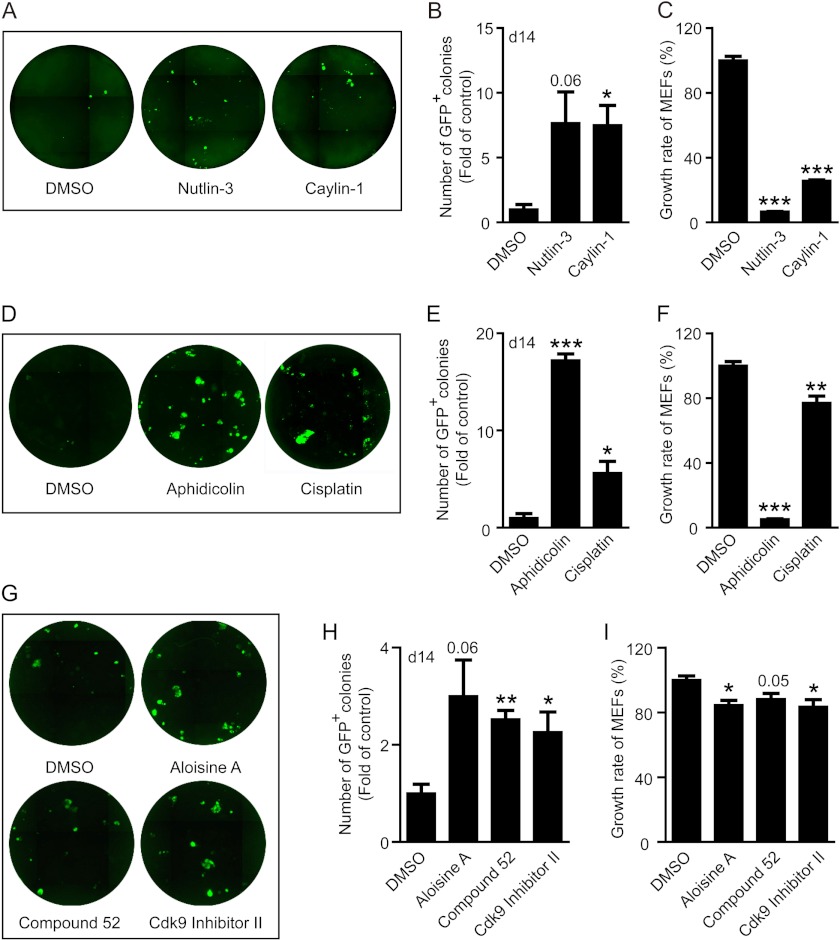
**Small molecules inhibiting cell proliferation improve iPS cell generation.** Various anti-proliferative agents were added into the 4 factor-mediated iPS generation system from day 3 to 8, and representative images and statistical analysis of GFP^+^ colonies are presented. Meanwhile, the growth inhibition of MEFs by these chemicals was tested in the same culture conditions as used in reprogramming and cell growth was monitored with FACS analysis. *A–C*, the effect of p53 activators nutlin-3 (10 μm) and caylin-1 (10 μm) on iPS cell generation (*A* and *B*) and MEF proliferation (*C*). *D–F*, the effect of chemotherapy drugs aphidicolin (600 nm) and cisplatin (300 nm) on iPS cell generation (*D* and *E*) and MEF proliferation (*F*). *G-I*, the effect of kinase inhibitors targeting cell-cycle machinery, including aloisine A (100 nm), compound 52 (100 nm), and Cdk9 inhibitor II (100 nm), on iPS cell generation (*G* and *H*) and MEF proliferation (*I*) (*, *p* < 0.05; **, *p* < 0.01; ***, *p* < 0.001, or stated otherwise).

To further test our hypothesis that hyperproliferation after viral transduction is unfavorable to iPS cell generation, we tested a variety of antiproliferative chemicals in the OSKM-mediated reprogramming system. To prevent inhibition of viral infection and iPS cell proliferation, all chemicals (at subtoxic level) were added after viral transduction and lasted only 5 days at the early stage of reprogramming (from day 3 to 8). Meanwhile, the growth inhibition of MEFs by these chemicals was carried out in the exact same culture conditions. Because activation of p53 is a well defined mechanism to inhibit cell proliferation, we first tested two p53 activators, nutlin-3 and caylin-1. Both effectively inhibited the growth of MEFs (Fig. 5*C*), but increased the number of GFP^+^ colonies (Fig. 5, *A* and *B*), suggesting that a temporary activation of p53 facilitates reprogramming. Next we tested several commonly applied chemotherapeutic drugs capable of inhibiting cell proliferation (Fig. 5*F*). One particular drug, aphidicolin, dramatically boosted iPS cell generation and another widely used compound, cisplatin, also enhanced reprogramming efficiency (Fig. 5, *D* and *E*). Finally we screened a number of inhibitors targeting cell cycle machinery, and identified several candidates that also facilitate iPS cell generation (Fig. 5, *G* and *H*), whereas inhibiting the growth of MEFs (Fig. 5*I*). Interestingly, compounds with better MEF growth inhibition also displayed better enhancement of reprogramming, such as aphidicolin (Fig. 5, *E* and *F*); whereas compounds like aloisine A, which inhibited the growth of MEF by <20%, only enhanced reprogramming by ∼2-fold (Fig. 5, *H* and *I*). In addition to the chemicals mentioned above, many of the recently reported small molecules that enhance reprogramming, such as VPA, BIX-01294, 5-azacytidine etc., are also antiproliferative (Table 3), apart from their reported roles in regulating epigenetic modification and pathway activities during reprogramming.

**TABLE 3:** **Small molecule enhancers on reprogramming and their effects on cell proliferation**

Small molecules	Molecular targets	Functions in reprogramming	Effects on cell proliferation
BIX-01294	G9a histone methyltransferase (G9a HMTase) inhibitor	Promote reprogramming of MEFs and neural progenitor cells using OK (47, 48).	Suppress cancer cell proliferation (49).
RG108	DNA methyltransferase inhibitor	Promote reprogramming using OK with BIX (47).	Displays antiproliferative properties against cancer cells with no detectable cytotoxic effects (50).
Bay K8644	l-Calcium channel agonist	Promote reprogramming using OK with BIX (47).	Depend on concentration towards mouse spleen lymphocytes (51).
Parnate	Lysine-specific demethylase 1 inhibitor	Promote reprogramming of human primary keratinocytes using OK (52).	Not directly addressed.
Kenpaullone	Inhibitor of GSK-3β, Cdk1/cyclin B, Cdk2/cyclin A, and Cdk5/p35	Promote reprogramming of MEFs without Klf-4 (53).	Delay cell cycle progression on MCF10 A cells (54).
Valproic acid	HDAC inhibitor	Promote reprogramming of MEFs using OSK or OSKM and human fibroblast using OS, OSK (55).	Suppress cell proliferation in various cell types (56, 57).
			Stimulate proliferation of hematopoietic stem cells (58).
Trichostatin A	HDAC inhibitor	Improve reprogramming of MEFs (55).	Inhibit cell proliferation in a variety of cancer cells (59, 60).
Suberoylanilide hydroxamic acid (SAHA)	HDAC inhibitor	Improve reprogramming of MEFs (55).	Inhibit of cell proliferation and induce cell cycle arrest (61–63).
5-Azacytidine	DNA methyltransferase inhibitor	Promote reprogramming of MEFs (55).	Inhibit cell proliferation of NIH-3T3 cells (64).
Dexamethasone	Synthetic glucocorticoid	Acts with 5-azacytidine to promote reprogramming (55).	Suppress EGF-stimulated proliferation of rat gastric epithelial cell. Suppress proliferation of C6 glioma cell and human bone marrow stromal cells (65–68).
Wnt3a conditioned medium	Provide Wnt signaling	Promote reprogramming of MEFs using OSK (69).	Promote proliferation of stem cells; continuous Wnt exposure resulted in a marked decrease in proliferation of MEFs (70–72).
CHIR99021	GSK3 inhibitor	Enable reprogramming of MEFs using OK (52).	Antiproliferative against several tumor cell lines (73).
A-83–01	Inhibitor for the TGF-β pathway	Promote reprogramming of neonatal human epidermal keratinocytes using OK (74).	No apparent affect on cell proliferation assessed in HM-1 cells. Block TGF-β-induced cell proliferation arrest (75, 76).
SB431542	TGF-β inhibitor	Promote reprogramming of human fibroblasts (52).	Stimulate proliferation of endothelial cells derived from embryonic stem cells (77).
PD0325901	MEK inhibitor		Inhibit proliferation of tumor cells (78).
Vitamin C	Alleviates senescence	Promote human and mouse fibroblast reprogramming (79).	Promote proliferation (79).
E-616452 (Repsox)	Inhibitor of the Tgfbr1 kinase	Promote reprogramming of MEFs in absence of Sox-2 (80).	Induce G_1_/S arrest (80).
Butyrate	Small-chain fatty acid	Promote reprogramming in the absence of either c-Myc or Klf4 (81).	Inhibit proliferation in various tumor cells (82).
LiCl	Affects several enzymatic activities	Enable reprogramming of MEFs with (17).	Induce apoptosis in cancer cells (83).

## DISCUSSION

The striking improvement of reprogramming efficiency by removing c-Myc from the Yamanaka factors is quite unexpected. Although it is generally accepted now that c-Myc is dispensable for iPS cell induction, the absence of exogenous c-Myc during reprogramming has also been reported to seriously affect the efficiency and kinetic of the process (4, 21). The Myc transcription factor family (c-, N-, and L-Myc) has well established roles in the control of cell cycle progression, cell immortalization, and tumor progression (12, 28). Unlike other transcription factors used for somatic cell reprogramming, such as Oct4 and Sox2, which have key roles in maintaining the pluripotent state, there is no direct evidence linking c-Myc to the development of stem cells in the peri-implantation stage embryo. The absence of an early developmental phenotype in c-*Myc* knock-out mice also confirmed this (29). Although Myc has well recognized roles in blocking cell differentiation and maintaining progenitor populations *in vivo* (30–32), it has also been reported to promote progenitor differentiation (33–35).

Early studies demonstrated the roles of c-Myc in reprogramming in several ways. One simple explanation is that Myc can maintain somatic cells in a proliferative state that might facilitate the retroviral-mediated transduction of reprogramming factors. Viral infection efficiency has long been associated with reprogramming efficiency. A recent study also demonstrated that serum starvation-induced cell cycle synchronization significantly improved viral infection efficiency and thus reprogramming efficiency (16). c-Myc is also an important regulator involved in cell senescence, which has also been demonstrated as a major roadblock against reprogramming (20, 36). c-Myc is required for sustained proliferation of primary MEFs (37), and it is down-regulated during senescence (38). Forced expression of c-Myc facilitates senescence escape (39). However, overexpression of c-Myc as well as other Yamanaka factors has also been reported to induce cellular senescence in IMR90 cells (36), indicating that many of the transcription factors have context-dependent effects on cell fate. Although Myc is not critical for *in vivo* ES cell development, ectopic Myc expression has been shown to relieve the dependence of mES cells for LIF *in vitro* (11). Myc has also been reported to silence genes associated with differentiation and modify the epigenetic signature in a global level, which might prime the reprogramming process (40).

However, our study demonstrated that ectopic expression of c-Myc might be detrimental to reprogramming. Removing c-Myc from the 4 factors resulted in a more than 10-fold increase in reprogramming efficiency. The iPS cells generated without c-Myc are genuinely pluripotent and have passed the most stringent tetraploid complementation test and generated a full-term iPS mouse. Although not fully understood at the present stage, this apparent discrepancy between our study and previous reports might due to our slightly modified iPS induction protocol. Earlier studies utilized a serum-based medium (mES medium) for the whole reprogramming process. In our protocol, 2 days (day 2) after viral infection (day 0), MEFs were reseeded onto 96-well plates pre-seeded with irradiated MEF feeders, supplemented with mES medium (DMEM supplemented with 15% FBS, to ensure the survival of feeder cells). At day 6, culture medium was replaced with knock-out serum replacement medium (knock-out-DMEM supplemented with 15% knock-out serum replacement), a serum-free medium. It has been reported that the c-Myc expression level in ES cells cultured in medium containing serum is much higher than those cultured in serum-free medium containing 2i (41). This is probably why c-Myc facilitates reprogramming in serum containing medium. The 2i in serum-free conditions renders Myc-Max complexes dispensable in the self-renewal of ES cells (42). The context dependence of c-Myc function might help to explain why it is detrimental to reprogramming in our system.

More interestingly, we found that forced expression of c-Myc led to a hyperproliferation state of the MEFs, which correlated negatively to the overall reprogramming efficiency. The relationship between somatic cell proliferation control and reprogramming efficiency has never been clearly defined. Various approaches targeting senescence, often accompanied by increased cell proliferation, have been implemented as a common strategy to enhance reprogramming by early studies (20, 36, 43, 44). A recent study also demonstrated that a high proliferation rate was required for establishment of human iPS cells (45). But many of the strategies that stimulate MEF proliferation also stimulate iPS or ES cell proliferation. Due to the lack of early markers for successful reprogramming, it is difficult to distinguish whether the high proliferation rate came from the starting somatic cells or the reprogrammed iPS cells. Because ES cells grow much faster than fibroblast, it is not surprising to find higher proliferation rate after successful reprogramming.

It is generally accepted that transcription factor-mediated reprogramming is an inefficient and gradual process. Although this process likely depends on rare and stochastic events, several critical steps such as the loss of differentiated cell characteristics, acquisition of a pre-pluripotent state, expression of key pluripotent genes, and emergence of the self-sustained pluripotent state are believed to be required for reprogramming (46). The initiation of reprogramming involves a global epigenetic and expression profile change. Whether such a change can be finished in one cell cycle or needs several cell cycles to be completed remains elusive. From our study, it seems that a normal proliferation of somatic cells is needed for retroviral infection. But after viral infection, keeping the somatic cells at a low proliferation rate is essential to reach high reprogramming efficiency. We speculate that the continuous rapid proliferation of somatic cells after viral infection will introduce more variations and dilute the epigenetic change that is necessary for successful reprogramming.

In conclusion, we think it is good to know that removing c-Myc from Yamanaka factors improves reprogramming efficiency without affecting the pluripotency of the resulting iPS cells. Our data also sheds new perspectives upon the role of somatic cell proliferation control in reprogramming and proposed a novel approach to enhance the generation of iPS cells by temporarily slowing down somatic cell proliferation at the initial stage of reprogramming. The detailed relationship between somatic cell cycle control and reprogramming warrants further investigation.

## Footnotes

3 The abbreviations used are:

iPS: induced pluripotent stem
MEF: mouse embryonic fibroblast
CFSE: carboxyfluorescein diacetate succinimidyl ester.
